# TOPIC: "The level of nurses' knowledge on occupational post exposure to hepatitis B infection in the Tamale metropolis, Ghana"

**DOI:** 10.1186/s12913-017-2182-7

**Published:** 2017-04-05

**Authors:** Kennedy Diema Konlan, Millicent Aarah-Bapuah, Joseph M. Kombat, Gifty Mary Wuffele

**Affiliations:** 1 Tamale Teaching Hospital, Tamale, Ghana; 2 Department of Nursing, University for Development Studies, School of Allied Health Sciences, Tamale, Ghana; 3 Department of Paediatrics, University for Development Studies, School of Allied Health Sciences, Tamale, Ghana; 4 Department of Midwifery, University for Development Studies, School of Allied Health Sciences, Tamale, Ghana

**Keywords:** Knowledge, Awareness, Vaccination status, Post exposure measures, Hepatitis B

## Abstract

**Background:**

Health care workers especially nurses in developing countries are at serious risk for infection from blood-borne pathogens particularly hepatitis B virus (HBV) — because of the high prevalence of such pathogens in many poorer regions of the world. Employers are required to establish exposure-control plans that include post exposure follow-up for their employees and to comply with incident reporting requirements.

This study assessed the level of knowledge and awareness of nurses in the Tamale Metropolis of Ghana on occupational post exposure measures to hepatitis B, and their risk of being infected. Hepatitis B vaccination status of nurses was also assessed.

**Methods:**

A cross-sectional study was conducted involving 108 nurses of varied categories who were selected by simple random sampling from west and central hospitals in the Tamale Metropolis of Ghana. Data was collected using a semi-structured questionnaire. SPSS version 16 was used for the analysis of data.

**Results:**

Ninety-four percent (94.4%) of the nurses considered themselves susceptible to occupational infection of HBV. About 23.4% were able to mention all the key elements of the post exposure management with 12.1% having adequate knowledge on post exposure prophylactic treatment against HBV. However, only 48 (44.4%) nurses have received hepatitis B vaccination. Thirty-six (75%) of those immunized had received three doses as required while the remaining had less than 3 doses. Some (38.9%) recap used needles before disposal and 30.2% do not decontaminate blood and body fluids before disposal.

**Conclusion:**

Nurses are aware of their risk of occupational exposure to hepatitis B but lack the requisite knowledge on post exposure management as well as measures that reduce the exposure. Nurses should familiarize with the principles of post exposure management as part of job orientation and on-going job training. Also, there is a need for a national policy on occupational safety and health which should include HB vaccination of health care workers as a requirement for appointment into the health service.

## Background

Health care workers in developing countries are at serious risk of infection from blood-borne pathogens — particularly hepatitis B virus (HBV), hepatitis C virus (HCV), and HIV — because of the high prevalence of such pathogens in many poorer regions of the world [12]. Theobald Owusoansah [14] reported that in Ghana, hepatitis B is the leading cause of infectious death, claiming the lives of thousands of Ghanaians each year [14]. In showing how serious the hepatitis B infection is in the country, Dongdem et al. [5] corroborated with these views in reporting that among blood donors at the Tamale Teaching Hospital. The age group with the highest number of donors (53.47%) was 20–29 years which also constituted the highest number of positive cases (69.35% of all positive cases) among voluntary donors [5].

According to World Health report [17], 2.5% of HIV/AIDS cases among health care workers and 40% of hepatitis B and C cases among health workers are the result of occupational exposure. Studies suggest that needle stick injuries pose the greatest threat to health care professionals (HCP) as these diseases are transmitted through blood. The risk of infection after exposure to infected blood varies by blood-borne pathogen. The risk of transmission after exposure to HIV-infected blood is about 0.3%, whereas it is estimated to be up to 100 times greater for hepatitis B virus (30%) and could be as high as 10% for hepatitis C virus [3]). It has also been reported that nurses experience the majority of needle stick injuries in the world including half of the exposures that occur in the US [2] and 70% of exposure that occur in Canada [3]. Needle stick injuries are the most common exposure to the hepatitis B virus infection) [13]).

Unlike developed countries, most developing countries may not have surveillance for occupational exposure to blood and body fluids which precludes estimation of the exact magnitude of such accidents. Available statistics probably underestimate the severity of the problem because many workers do not report exposure to hepatitis B virus infection or the risk of exposure. Therefore making it difficult to know exactly how serious the problem is or how well prevention programmes work [3]. Failure to report these injuries may compromise appropriate post-exposure management, including Post-Exposure Prophylaxis (PEP) for HIV and hepatitis B virus, and assessment of occupational hazards and preventive interventions. The absence of post-exposure prophylaxis (PEP), lack of knowledge of the efficacy of PEP for prevention, an attitude that health care workers (HCWs) are careless or to blame for their own injuries, and lack of follow-up and workers’ compensation are all reasons HCWs do not report injuries [16]. Some nurses also see these injuries as part of the job and therefore accept them as normal consequences of their job. The commonest reaction after an exposure among nurses is to wash the site and squeeze out blood if the accident involves a needle prick. Only a few will go a step further to report or find out the sero status of the source patient [9].

Reports of Gurubacharya et al. [7] indicate that knowledge of health care workers about the risk associated with needle-stick injuries and use of preventive measures was inadequate. Results from their survey showed that 4 and 61% of nurses and paramedics respectively, were unaware of the fact that hepatitis B and hepatitis C can be transmitted by needle stick injuries but were aware of the fact that HIV can be transmitted by needle stick injuries [7].

Also a study by Bilski and Wysocki, [1] on the level of knowledge of post exposure prophylaxis of blood-borne infection at work place observed in nurses showed inadequate knowledge as between 21.6 to 29.6% of nurses could not list any principle of post exposure prophylaxis to hepatitis B. They however showed best knowledge of principles for HIV and worse for HCV [1].

The risk for transmission of HBV is reduced by immunization against hepatitis B, which is 90 to 95% effective. A research conducted in a teaching hospital in Nigeria to assess the acceptance of hepatitis B vaccine by workers revealed that workers with the highest possibility of, and exposure to hepatitis B infection within the hospital setting showed the greatest apathy to the vaccination programme [6].

Ghana has not done much regarding health care personnel occupational exposure to blood-borne pathogens. Assessing the level of knowledge and awareness of nurses under Ghana Health Service in the Tamale Metropolis of their risk of being infected with HBV and post exposure measures provided insight into the problem.

## Methods

### Study design

A descriptive cross-sectional study involving nurses of various categories working at Tamale west and Central hospitals within the Tamale Metropolis of Ghana. The cross sectional study design was necessary because data was collected from study participants once and inferences made on their attitude towards exposure and the post exposure measures usually adopted during risk. The study assessed nurses’ level of knowledge on post exposure measures instituted, nurses vaccination statues and the nurses knowledge on the various means of transmission of the virus.

### Population and sample

The study specifically targeted nurses working in the hospital setting who perform exposure-prone procedures (EPP). A sample size of one hundred and eight (108) nurses was drawn from the two hospitals by simple random sampling. A sample frame of the two hospitals was made. Participants were blindly handpicked from a basket containing all the names of nurses within the two hospitals. This sample was determined using the sample size determination formula by Israel Glenn D. in the paper sampling the Evidence of Extension Program Impact [8]. The response rate was 100%.

This sample was determined using the following sample size formula by Israel Glenn D.


Where nh = sample size, *N* = target population which is 148, and e is the margin of error taken as 5% (0.05) with a confidence level of 95%.

### Data collection techniques

Semi-structured Self-administered questionnaire was given to respondents who consented to the study to complete and submit within a specified period. The questionnaire was pre-tested with twenty nurses at the Seventh-day Adventist Hospital within the Tamale metropolis.

### Data analysis methods

The data was first assessed for appropriateness and completeness before entered in to the software for the analysis. Statistical package for social sciences (SPSS) version 16 was used to analyse the data and results presented using simple descriptive statistics. The descriptive statistics used were simple proportions as views were expressed in percentages.

### Ethical approval

Approval was obtained from the authorities of Tamale West and Central hospitals and verbal consent obtained from the participants before the implementation of the study. Respondents also gave consent for publication of the research findings after it was duly explained to them.

## Results

### Demographic characteristics of respondents

Respondents were 65.7% registered general nurses, enrolled nurses (20.4%), midwives (7.4%) and other cadre of nurses (6.5%) who have specialized in various fields (Tables 1 and 2).

**Table 1:** Distribution of demographic characteristics of respondents

Variables	Distribution	Frequency	Percent
Age distributions	20–30	75	69.4
	31–40	20	18.5
	41–50	5	4.6
	51–60	8	7.4
Religious status	Islam	48	44.4
	Christianity	56	51.9
	Traditional	4	3.7
Sex	Male	61	56
	Female	47	44

**Table 2:** Distribution of category of nurses and duration of work experience

Variables	Distributions	Frequency	Percent
Category of nurse	Enrolled nurses	22	20.4
	Registered general nurses	71	65.7
	Midwives	8	7.4
	Specialized	7	6.5
Duration of work experience	<5	62	57.4
	6–10	27	25.0
	11–15	9	8.3
	16–20	2	1.9
	>20	8	7.4

### Awareness of risk of occupational exposure to hepatitis B

Majority (94.4%) of nurses are occupationally susceptible to HBV infection. Awareness of the risk of infection influenced staff performance (72.2%) at duty. Fifty-nine (59.3%) of those whose performance was influenced by awareness of risk of infection stated that they were cautious to avoid injuries/accidents, (7.4%) avoided touching infected patients while 5.6% stated that they did not conduct task very well. Majority (91.7%) of the respondents knew that hepatitis B is caused by a virus. However, only 14.8% of respondents had full complement of knowledge on substances that may contain the hepatitis B virus or the means of transmission of the virus. Majority (57.4%) of respondents were aware that HBV may be occupationally spread via contact with infected blood and blood product. Assessing major risk of exposure revealed; 57% needle stick injury, 38.3% splash of blood/body fluids into mucous membranes and broken skin and 4.7% mentioned injuries occurring from scalpel blades.

### Knowledge on treatment after exposure

Small proportion (12.1%) had accurate knowledge of the PEP treatment against hepatitis B which is hepatitis B Immunoglobulin (HBIg) and hepatitis B vaccine with only 2.8% knowing of the use of Hepatitis B Immunoglobulin (HBIg.) The results also indicate that 23.4% had knowledge of all the key elements of the post exposure management indicated by Occupational Safety and Health Authority (OSHA). Some respondents (15.9%) omitted the immediate step of allowing the site to bleed and washing with soap and water, as 5.6% performed only the immediate step which is to allow bleeding and washed. Some nurses (17.8%) washed and reported and 7.5% tried to arrest bleeding or use disinfectants to clean the site.

Many of the respondents (64.8%) usually report exposures. The major reason for not reporting among the remainder was the fact that no measures are usually taken following a report as indicated by 21/38 respondents. Other reasons stated included not knowing who to report to, and nurses considering these accidents as part of their job as in the case of 21.1% respondents who do not report.

Most (69.4%) nurses know that PEP should be taken as early as possible for effectiveness, however, 19.4% did not know how early it should be taken and 3.7% stated it could be taken at most within a month. Most (87%) of the nurses had never attended a refresher training on PEP.

### Hepatitis B immunization status of nurses

The study also revealed that only 44.4% of nurses had taken the vaccine against hepatitis B. Among the 48 immunized persons, 75% had received three doses as required. Also some (75%) acquired the vaccine through their own efforts, while for 22.9%, it was provided by the employers. Vaccine affordability (50.8%) and accessibility (16.9%) were the major reasons for non-immunization among the unimmunized staff. Others (20.3%) gave other reasons centred on procrastination for example, “I am yet to go for it” or “I have not yet decided to go for it” while 10.2% thought it was not necessary because they followed universal precautions. Fear of injection by needles was the least reason for non-immunization.

### Reducing the risk of occupational exposure

Fifty six (51.9%) respondents described the supply of gloves in their units as adequate. Fifty six percent (56.5%) of respondents used gloves for only procedures involving contact with blood as against 43.5% who used gloves for all nursing procedures. Majority of respondents (67.6%) routinely practice hand washing before and after every procedure, and 31.5% practiced hand washing after every procedure. The results also show that some nurses (58.3%) do not recap used needles before disposal into a safety box while 38.9% recap needles before disposal into a safety box. Many respondents (70%) decontaminate blood, body fluids, and stool before disposal and 12.3% go further to decontaminate the receptacle or the waste container.

## Discussion

This study assessed the level of knowledge and awareness of nurses in the Tamale Metropolis on occupational post exposure measures to hepatitis B, and the risk of being infected. Most nurses are very much aware that they are occupationally exposed to hepatitis B as indicated by the majority. Similarly, Lemessa [9], reported 97.2% awareness of occupational exposure to blood borne pathogens among HCW in Army force Referral and teaching hospital, Addis Ababa, Ethiopia. This high level of awareness has direct positive influence on performance of duties making nurses more cautious in order to avoid injuries/accidents. It also has negative influence in some nurses because they tend to avoid touching patients especially those known to be infected with the disease. Also, some nurses are not able to conduct task very well which subsequently affect the quality of care they render to clients. This finding is consistent with the results of a study conducted in Uganda among health care workers by [4].

Majority of the nurses showed adequate knowledge on causes of hepatitis B but had considerable gaps in their knowledge about substances that can contain hepatitis B virus. Respondents do not know which substances or procedures that universal precautions apply to and by extension, substances that pose a threat to hepatitis B infection.

Lack of knowledge about the mode of occupational transmission poses a danger to nurses and patients as well. Only 57.4% of respondents rightly stated contact with infected blood and body fluids as the mode of occupational transmission among nurses. Most respondents (78.7%) know that needle stick injury (NSI) and splash of blood and body fluids into mucous membranes/non intact skin poses exposure risks. Worrying is the fact that 7.4% did not know that splash of blood into mucous membranes/non intact skin poses a risk to the hepatitis infection, and 3.8% did not consider NSI to pose an exposure risk. This is worrying because such nurses are not likely to identify the risk even when exposed thereby limiting the chance of reporting and receiving the post exposure prophylactic treatment they are expected to receive. This generally put such cadre in to greater risk than nurses who identified the risk. These results are consistent with the findings of Gurubacharya et al. where 4% of nurses were unaware that hepatitis B and C could be transmitted via infected needles [7]. The results also indicate that NSI poses the greatest exposure threat to HBV infection. The most frequently experienced exposures were NSI followed by splashing. These exposures were rare, occurring less than twice a year in most cases. It is worth noting that it takes a single exposure to cause an infection if prompt measures are not taken after that exposure, hence though rare, exposures should not be taken for granted.

Most nurses know of post exposure prophylaxis (PEP) but concerning hepatitis B specifically, only 9.3% know that hepatitis B vaccine is used as prophylaxis and only 3 persons mentioned hepatitis B immunoglobulin (HBIG) which is sometimes given together with the hepatitis B vaccine as PEP- a demonstration of inadequate knowledge in PEP for hepatitis B virus infection. Concerning steps taken following an accidental Needle Stick Injury (NSI), respondents showed gross knowledge deficits as they could not mention all the key elements in the post exposure management principles according to Occupational Safety and Health (OSH), Canada, which include allowing the area to bleed, immediate washing with water and soap, reporting the incidence, laboratory screening of both patient and the nurse, taking PEP (HBIG/Hepatitis B vaccine). Similarly Dieleman et al. [4] noted that the most common reaction after injuries among 79% of respondents in a survey conducted in Uganda is to wash the wound. Only on very few occasions (11%) was the patient tested, and only one person went for post-exposure prophylaxis (PEP).

It was revealed that most nurses report their exposures or injuries to the nurse in charge, or to a doctor, while others do not report injuries due to less faith in the system that something will be done, and some do not know who to report to. These findings share similarity with results of a study by Mbaisi et al. [10] where 52.5% reported the incident of percutaneous injury and source patient identified in 91.5% of cases. Health institutions need to appoint PEP officials who should be equipped to function adequately. Employees should be orientated to the reporting systems of the facilities. Not reporting an exposure incident means that such victims are unlikely to receive PEP and cannot be duly compensated if they develop an infection.

In contrast to a study [11] where only 18% of nurses understood that PEP need to be initiated immediately to be effective, most nurses in this study have adequate knowledge about PEP to be taken as early as possible (between 24 h and 1 week) for effectiveness. PEP should be taken as early as possible because the longer you wait, the less effective PEP may become. Also, most nurses have never attended any refresher training on PEP after the basic training from school. This means that the hospitals’ training programmes on PEP are inadequate.

Tests show that about 90 to 95% of vaccinations of healthy people will result in the development of resistance against hepatitis B [15]. At present, vaccination is the surest way to avoid acquiring hepatitis B as an occupational disease. Compared to the 68% vaccination uptake among nurses studied in Tshwane, South Africa, only 44.4% nurses in this study have received hepatitis B vaccine. Some have completed the full schedule of immunisation while others are yet to. The sero conversion status or post vaccination titres of those who have received all three doses have not been verified by this study. Nurses intimated that the most effective preventive measure against occupational hepatitis B is immunization.

Another way of occupationally acquiring the disease is when the nurse does not use protective materials like gloves. Most nurses in the hospitals studied use gloves for procedures involving blood but not for all procedures. The supply and availability of gloves in the work environment was deemed inadequate. It is important that nurses use gloves for all procedures that might expose them to even dried body fluids in used bed linen or receptacles since the virus can stay on dry surfaces for up to a week. Besides, one might be unaware of bruises/cuts on the hands of clients or the service provider.

Hand washing is the simplest and most important preventive measure which if not done or done well, can promote cross infection from patient to patient or the service provider. Most nurses are abreast with knowledge of how to dispose of blood, body fluids, secretions as well as needless and sharps according to the infection control guidelines. This is evidenced by decontamination and correct hand washing practices. Decontamination reduces the number and infectivity of microbes such that any accidental splashing during disposal will pose minimum risk. Similarly [11] found that 67% of nurses recapped needles after use as some (38.9%) nurses in this study. Also, in this study 2.8% stated that they put needles in the dust bin instead of the approved safety boxes. WHO recommends that needles should never be recapped, bent, or disassembled and should be disposed of in safety boxes.

The major limitation for this study was that nurses were not tested for the hepatitis B surface antigen and the researchers mainly relied on the information supplied by the nurses on their vaccination status. This could have been influenced by recall bias as most participants could have forgotten their vaccination status. Also the major tool used for data collection was self-developed. No scientifically proven standard tool was used to assess level of knowledge. Future research will focus on developing and using standardised tool in measuring knowledge on post exposure risk of nurses.

## Conclusion

Nurses working in the Tamale Metropolis are aware of their risk for occupational infection to hepatitis B virus. They however lack the requisite amount of knowledge about post exposure management as well as prevention of occupational exposures. This study has also revealed that most of the nurses working in Ghana Health Service facilities in the Tamale Metropolis have not been immunized against hepatitis B virus infection.

### Recommendations

Training and education in injection safety, prevention of sharps injuries, and universal precautions must be incorporated as part of on-going job training and refresher programmes.

Health Care Personnel especially nurses should familiarize with the principles of post exposure management as part of job orientation and on-going job training.

## Abbreviations

AIDS: Acquired immune deficiency syndrome
ANA: American Nurses Association
BBVI: Blood borne viral infections
CCOHS: Canadian centre for occupational health and safety
CDC: Centre for disease control
CHAG: Christian Health Association of Ghana
CHPS: Community-bases health planning and services
EPP: Exposure prone procedures
GHS: Ghana health service
HB: Hepatitis B
HBIG: Hepatitis B immuno globulin
HBV: Hepatitis B virus
HCP: Health care personnel/Professionals
HCV: Hepatitis C virus
HCW: Health care workers
HIV: Human immune virus
NSI: Needle stick injury
OHS: Occupational health and safety
OSHA: Occupational safety and health authority
PEP: Post exposure prophylaxis
PPE: Personal protective equipment
SPSS: Statistical package for social scientists
WHO: World Health Organisation
